# New perspectives on endocrine therapy suitability for hormone receptor-positive metastatic breast cancer in clinical practice

**DOI:** 10.1016/j.breast.2026.104831

**Published:** 2026-06-04

**Authors:** Hope S. Rugo, Giuseppe Curigliano, David W. Cescon, Frédérique Penault-Llorca, Nadia Harbeck, Seock-Ah Im, Yeon Hee Park, Carlos Barrios, Shanu Modi, Sara M. Tolaney, Mafalda Oliveira

**Affiliations:** a City of Hope Comprehensive Cancer Center, Duarte, CA, USA; b European Institute of Oncology, IRCCS, Milan, Italy; c University of Milano, Milano, Italy; d Princess Margaret Cancer Centre, University Health Network, Toronto, ON, Canada; e Centre Jean Perrin, Université Clermont Auvergne, INSERM, France; f Breast Center, Department of Obstetrics and Gynecology and Comprehensive Cancer Center Munich, Ludwig Maximilians University Munich University Hospital, Munich, Germany; g Cancer Research Institute, Seoul National University Hospital, Seoul National University College of Medicine, Seoul, South Korea; h Division of Hematology-Oncology, Department of Medicine, Samsung Medical Center, Sungkyunkwan University School of Medicine, Seoul, South Korea; i Latin American Cooperative Oncology Group, Porto Alegre, Brazil; j Department of Medicine, Memorial Sloan Kettering Cancer Center, Weill Cornell Medical College, New York, NY, USA; k Brigham and Women's Hospital Dana-Farber Cancer Institute, Boston, MA, USA; l Vall d’Hebron Hospital and Vall d’Hebron Institute of Oncology, Barcelona, Spain

**Keywords:** Endocrine therapy, Metastatic breast cancer, Expert opinion, Endocrine resistance

## Abstract

Hormone receptor-positive/HER2-negative advanced breast cancer (ABC) is a heterogeneous and dynamic disease. Endocrine therapy (ET) + cyclin-dependent kinase 4/6 inhibitors remain the standard-of-care first-line therapy for ABC. However, the treatment landscape is rapidly evolving as our understanding of the complex biology underlying this common subtype advances. Predicting how a patient's cancer might respond to ET across lines of therapy and understanding optimal sequencing in clinical practice are key unmet needs.

A range of established and emerging clinical characteristics and biomarkers, including endocrine receptor expression, presence of specific mutations (e.g., *ESR1*, *PIK3CA*), and visceral disease, are currently used to guide treatment decisions. However, international guidelines have variable definitions of ET resistance and sensitivity, making delivery of individualized care in clinical practice challenging.

Considering this unmet need and leveraging the existing evidence for both prognostic and predictive markers of therapeutic response, we propose that idea of ET suitability be used as a complement to ET resistance and sensitivity. We consider ET suitability to be the clinical assessment of whether a patient could benefit from ET, where benefit is defined not solely by tumor response but by a clinically relevant constellation of characteristics and markers possibly predicting the durability of disease response and symptom control.

Several unresolved questions remain regarding issues such as disease heterogeneity, optimal treatment sequencing, and biomarker precision, but further work and ongoing studies will help to support the evolution of guidelines and provide clarity around the effective application of this quickly developing field to daily clinical practice.

## Highlights

• HR+/HER2− advanced breast cancer (ABC) is a heterogeneous and dynamic disease.
• Predicting response of HR+/HER2− ABC to endocrine therapy (ET) remains a challenge.
• ET sensitivity is often used to guide decisions in the clinic, but definitions vary.
• We propose the concept of ET suitability as a complement to ET sensitivity.
• ET suitability defines benefit using a clinically relevant constellation of markers.

## 1 Introduction

The landscape of endocrine therapy (ET) for hormone receptor (HR)-positive/human epidermal growth factor receptor 2 (HER2)-negative advanced breast cancer (ABC) is rapidly evolving as our understanding of the complex biology underlying this common subtype advances. Currently recommended endocrine agents include aromatase inhibitors, selective estrogen receptor modulators, and selective estrogen receptor degraders (SERDs) [[1], [2], [3], [4], [5], [6]]. Alongside approved and established ET-based regimens, several are under clinical/preclinical investigation, including a number of novel SERDs and complete receptor antagonists, selective estrogen receptor covalent antagonists, and proteolysis-targeting chimeras [[7], [8], [9], [10], [11], [12], [13]]. Although endocrine agents combined with inhibitors of cyclin-dependent kinase 4/6 (CDK4/6) inhibitors constitute the backbone of first-line ET-based approaches, endocrine agents are also combined with other targeted therapies, e.g., phosphatidylinositol-3-kinase (PI3K), Akt serine/threonine kinase (AKT), and/or mechanistic targeting of rapamycin (mTOR) inhibitors [[1], [2], [3], [4], [5], [6]].

As the available treatment options for HR-positive/HER2-negative ABC continue to expand, it has become increasingly important to identify which patients will benefit from specific strategies at each line of treatment. Accordingly, identifying appropriate biomarkers and clinical factors to support clinical decisions has become a focus of research and clinical development, with a wealth of data becoming available over recent years. Treatment algorithms now integrate these data, highlighting key decision points integrated with clinical and biological patient characteristics, such as prior therapies, disease-free interval, the presence of visceral crisis, and molecular alterations of interest [[1], [2], [3], [4], [5], [6],^14^].

Despite research and ongoing expert discussions in the field, predicting whether a patient's tumor remains sensitive to ET across increasing lines of therapy and integrating emerging biomarkers into treatment decisions remain core clinical challenges. ​There remains considerable need to better distinguish patients who may continue to derive benefit from ET-based regimens from those patients who may not.

This commentary will explore current evidence supporting ET sensitivity and highlight the utility of ET suitability as a clinical practice-focused concept. Through this approach, we hope to provide insights into the evolving challenge of establishing how ET sensitivity and suitability may both support clinical practice now and in the future.

## 2 Defining ET resistance, sensitivity and suitability

The most broadly used definitions of ET response have been iteratively shaped by the European School of Oncology-European Society for Medical Oncology (ESO-ESMO) international consensus guidelines for advanced breast cancer (ABC guidelines). The development of these definitions provides insight into the changing needs of the clinical community and newly emerging data.

### 2.1 ET resistance

**Fig. 1:**
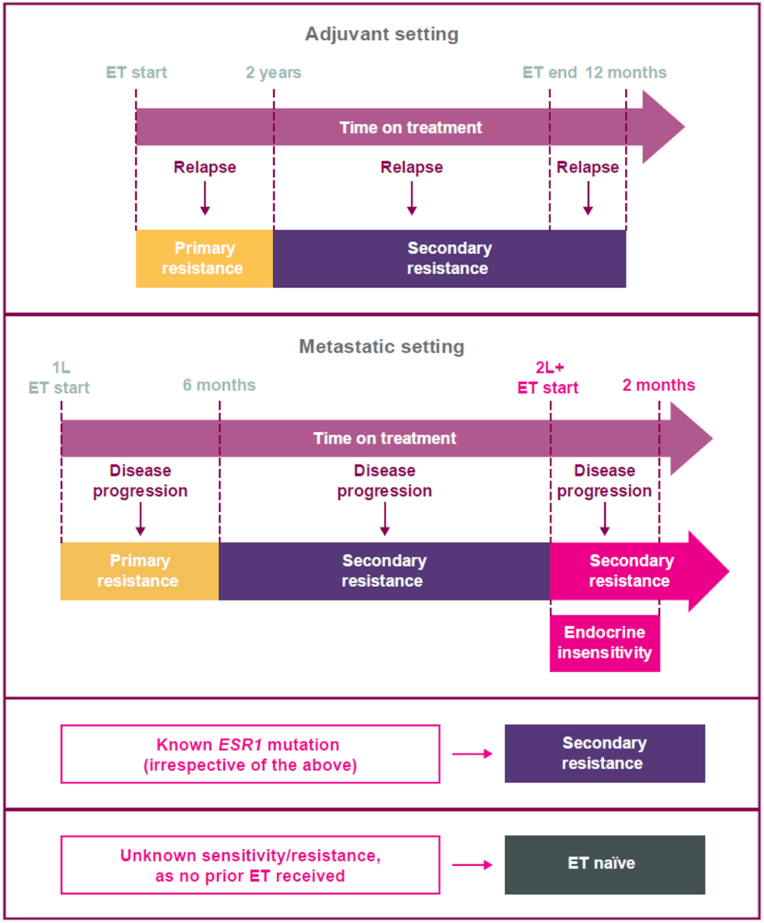
Guideline definitions of ET resistance and insensitivity. Elements in pink indicate updates made in ABC6/7 [4] vs ABC5 (ESO-ESMO) [15] guidelines. 1L, first-line; 2L+, second- and subsequent lines; ABC5, 5th ESO-ESMO international consensus guidelines for advanced breast cancer; ABC6/7, 6th and 7th International consensus guidelines for the management of advanced breast cancer; ESMO,European Society for Medical Oncology; ESO, European School of Oncology; *ESR1*, estrogen receptor 1 gene; ET, endocrine therapy. (For interpretation of the references to colour in this figure legend, the reader is referred to the Web version of this article.)

In 2020, the ABC5 guidelines defined primary ET resistance as relapse while on the first 2 years of adjuvant ET, or disease progression within the first 6 months of first-line ET for ABC (Fig. 1) [15]. Secondary (acquired) resistance was defined as relapse while on adjuvant ET after the first 2 years, relapse within 12 months of stopping adjuvant ET, or disease progression after the first 6 months of first-line ET for ABC after the first 6 months (Fig. 1) [15]. Although these definitions were derived from expert opinion rather than a study, their prognostic significance has been validated in clinical trials [16,17].

In mid-2024, the 6th and 7th International consensus guidelines for the management of ABC (ABC6/7) expanded the previous ABC5 definitions to incorporate recent developments in the treatment landscape of HR-positive/HER2-negative ABC (Fig. 1) [4]. The definition of primary ET resistance remained the same as in ABC5, but clarified that it applies regardless of prior CDK4/6 inhibitor. Secondary resistance was defined as relapse while on adjuvant ET after the first 2 years, or disease progression after the first 6 months on first-line, or after any duration on second- or subsequent-line ET for ABC. Finally, known estrogen receptor 1 (*ESR1*) mutations are included as an independent predictive factor for secondary ET resistance. The guidelines note that the definition of secondary resistance remains unaffected by therapy with inhibitors of CDK4/6, mTOR, PI3K, or other combination drugs. The ABC8 guidelines are currently under development following a meeting at the end of 2025.

The ESMO Clinical Practice Guidelines [5] currently define ET resistance in accordance with ABC5, while the American Society of Clinical Oncology (ASCO) and NCCN® Clinical Practice Guidelines in Oncology (NCCN Guidelines®)​ do not currently clearly define ET resistance, although the terms “endocrine refractory/resistant” are determined by clinical judgement [[1], [2], [3],^6^,^14^].

### 2.2 ET sensitivity

ET sensitivity is not defined in detail in any of the above guidelines [[1], [2], [3], [4], [5], [6],^14^,^15^]. However, ABC6/7 notes that in cases where a patient is ET-naïve, sensitivity or resistance to ET is unknown and patients can be considered ET sensitive until proven otherwise [4]. Furthermore, the ESMO guidelines use ET-naïve and ET sensitivity (undefined) alongside one another in the treatment algorithms for HR-positive/HER2-negative breast cancer. In the absence of known primary/secondary resistance and/or prior treatment with ET, a patient may be considered to have ET sensitive disease [4,18].

Unique among published guidelines, endocrine insensitivity is defined in ABC6/7 as disease progression during the first 2 months of second- or subsequent-line ET for ABC, where no additional ET-based approaches are likely to result in clinically meaningful benefit. When endocrine insensitivity arises in clinical situations, NCCN guidelines refer to the disease as endocrine refractory and recommend systemic chemotherapy or other non-ET-based targeted therapies [19]. As such, the terms ‘insensitive’ and ‘refractory’ are related; however, there is no detailed formal definition of refractory disease in currently published guidelines [20]. Importantly, ABC6/7 specifically notes that endocrine resistance is a continuum and ET resistance/insensitivity definitions are relative and the most relevant for clinical trials [4].

### 2.3 Limitations of existing definitions of ET response

Although the existing definitions of ET response constitute a useful tool to assist in clinical trial selection and evaluation of clinical trial data [16], they have several limitations. There is a lack of guidance regarding practical application​ in the clinic, and ascertaining the level of resistance of a given tumor with precision is challenging [4]. Existing definitions are mainly based on time on treatment without accounting for disease heterogeneity or the context of disease progression and while broadly applicable to ET when used as a single agent their application to combination therapies such as CDK4/6 inhibitors is less clear. The existing definitions therefore do not capture the evolving therapeutic landscape and growing knowledge about markers and mechanisms of ET response, nor are they easily applied to daily clinical practice​.

### 2.4 ET suitability

Given this, there is a need to give more weight to the concept of “ET suitability” as a complement to ET sensitivity, and to better understand how it applies to clinical practice. Here, we define ET suitability as the clinical assessment of whether a patient could benefit from ET with or without a targeted agent over other treatment options, where benefit is defined not by tumor response but by durability of disease response/stable disease/symptom control; in effect, ‘ET suitable’ defines the state in which is it clinically reasonable to select ET-based therapy for a patient based on a holistic view of their clinical and biological markers. For the purposes of this review, we define ET sensitivity as the degree to which a tumor responds to treatment with ET-based therapy. Although ET sensitivity remains relevant when assessing clinical trial data, here we will primarily focus on determining *suitability* for further lines of ET-based treatment within the context of clinical decision-making.

## 3 Role of clinically established markers on suitability for ET

**Fig. 2:**
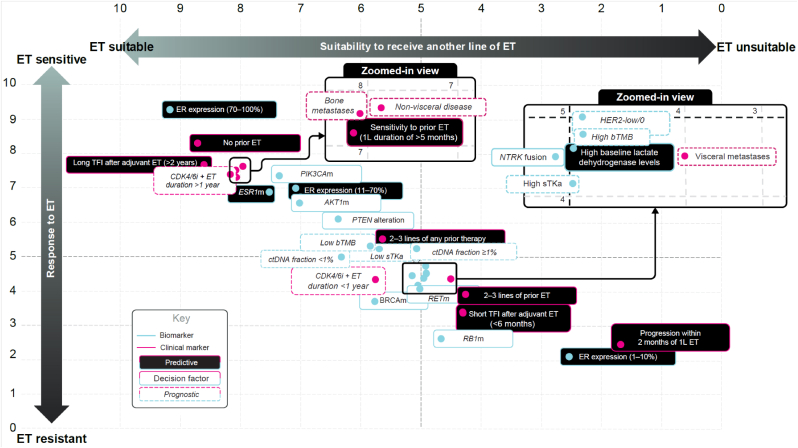
Expert assessment of markers underlying ET suitability and ET sensitivity for consideration in treatment sequencing in HR-positive/HER2-negative ABC. The grid shows a visual representation of individual markers plotted against their function underlying suitability to receive another line of ET (y-axis) and response to ET (x-axis) in clinical practice; placement of each marker against the x and y axes is based on published literature and author opinion. The authors emphasize the importance of considering markers holistically, rather than in isolation, and consider that some markers (e.g., visceral crisis) supersede others in guiding treatment decisions with ET. 1L, first-line; ABC, advanced breast cancer; *AKT1*, Akt serine/threonine kinase 1; *BRCA*, breast cancer gene; bTMB, blood tumor mutational burden; CDK4/6i, cyclin-dependent kinase 4/6 inhibitor; cfDNA, circulating free DNA; ER, estrogen receptor; *ESR1*, estrogen receptor 1; ET, endocrine therapy; Fulv, fulvestrant; HER2, human epidermal growth factor receptor 2; HR, hormone receptor; m, mutation; *NTRK*, neurotrophic tyrosine receptor kinase; *PIK3CA*, catalytic subunit alpha of phosphatidylinositol-3-kinase; PARPi, Poly (ADP-ribose) polymerase inhibitor; *PTEN*, phosphatase and tensin homolog; *RB1*, retinoblastoma 1; *RET*, rearranged during transfection; sTKa, serine threonine kinase a; TFI, treatment-free interval; TRKI, tropomyosin receptor kinase inhibitor.

A range of different biomarkers and clinical factors, currently acknowledged as key decision points in published guidelines [[3], [4], [5], [6]], form the basis of current clinical decision-making (Table 1) [16,17,[21], [22], [23], [24], [25], [26], [27], [28], [29], [30], [31],^33^,^34^,^36^,^37^,[39], [40], [41], [42],[46], [47], [48], [49], [50], [51], [52]]. It is important at this stage to note the distinction between prognostic and predictive markers of therapeutic response. Prognostic markers relate to the overall disease outcome expected in a patient, and predictive markers to the likelihood of response to a particular therapy ​ [53,54]. Prognostic and predictive markers can be used together with other decision factors (including patient preference and the availability of therapies) to estimate how a patient may respond to ET-based therapy. These markers can be conceptualized as a 2D-framework (Fig. 2) that visually maps a marker's association with ET sensitivity (the relationship between a marker and tumor response to ET) versus ET suitability (the impact of a marker on the degree or likelihood to which a patient may benefit from ET over other treatment options) based on a combination of published data and expert opinion. Accordingly, here we will report on the published data that support the role of each marker in determining ET sensitivity, and comment on how this relates to ET suitability.

**Table 1:** Established markers of ET suitability. Evidence strength graded using ESMO methodology.

Marker	Study type	Key evidence to support a role in influencing ET suitability	Evidence strength^a^
Estrogen receptor expression	Exploratory analysis of Phase I study	• High baseline estrogen receptor α pathway activity was a molecular predictor of response to giredestrant in the second- or third-line setting, irrespective of *ESR1* mutation status, indicating that it is a marker for ET sensitivity [21]	III
	Pilot clinical trial	Patients with higher estrogen receptor expression per^18^F-fluoroestradiol positron emission tomography/computed tomography (standardized uptake value ≥ 2) had prolonged survival on ET [22]	III
	Retrospective study	Patients with 71–100% estrogen receptor expression by immunohistochemistry showed an improved PFS versus those with a lower score [23]	IV
Disease-free interval after prior ET	Retrospective study	Sensitivity to prior ET is a biomarker for sensitivity to fulvestrant; patients with a duration of ≥25 months for prior adjuvant ET and ≥5 months for prior first-line ET reached a longer PFS on fulvestrant [24]	IV
Visceral crisis	Meta-analysis of four Phase III RCTs	A higher incidence of visceral relapse and specifically liver metastases was noted in the primary ET resistance cohort versus the endocrine-sensitive and secondary ET resistance cohorts A higher incidence of non-visceral relapse, and specifically bone metastases, was noted in the endocrine-sensitive cohort versus the primary and secondary ET resistance cohorts [16]	I
	Meta-analyses of 14 Phase III RCTs	ET monotherapy as either first- or second-line treatment improved survival outcomes for patients with non-visceral versus visceral disease [25]	I
	Phase II RCT	In the RIGHT Choice Phase II RCT, ribociclib + letrozole/anastrozole + goserelin was superior to the investigator's choice of combination chemotherapy in patients with clinically aggressive disease; however, subgroup analysis indicated that the degree of benefit was less in the presence of visceral crisis [26]	II
	Phase IV RCT	In the PADMA Phase IV RCT, palbociclib + ET was superior to chemotherapy ± maintenance ET in terms of improvements in TTF (hazard ratio 0.46; 95% CI 0.31–0.69; *p* < 0.001) and PFS (hazard ratio 0.45; 95% CI 0.29–0.70; *p* < 0.001) in patients with previously untreated HR-positive/HER2-negative ABC and an indication for chemotherapy [27]	I
	Phase II RCT	In the ABIGAIL Phase II RCT, abemaciclib + ET was superior to chemotherapy + maintenance ET in terms of higher early overall response rate in patients with previously untreated HR-positive/HER2-negative ABC with ≥1 feature of aggressive disease [28]	II
HER2 status	Retrospective analysis of Phase II clinical trial	In patients receiving first-line ET monotherapy, the objective response rate was worse in patients with HER2-low versus HER2-zero tumors (N = 233) [29]	III
	Meta-analysis of 12 retrospective studies	Shorter PFS with CDK4/6 inhibitors + ET was noted in patients with HER2-low versus HER2-zero tumors across lines or in the first-line and with palbociclib + ET in the second or subsequent-line (N = 3567) No significant differences in PFS, though, with first-line palbociclib + ET or CDK4/6 inhibitors + ET in the second or subsequent lines No significant differences noted for OS or objective response rate [30]	IV
	Subgroup analysis of Phase 3 RCT	No effect of HER-low status on PFS with elacestrant versus aromatase inhibitor + fulvestrant [31]	I
Genomic alterations			
*ESR1* mutations	Phase III RCT	Patients with emergence of *ESR1* mutations showed significantly improved PFS following a switch in first-line treatment to camizestrant + CDK4/6 inhibitor compared to patients who remained on aromatase inhibitor + CDK4/6 inhibitor (hazard ratio 0.44; 95% CI 0.31–0.60; *p* < 0.0001) [32]	I
	Meta-analysis of four Phase II/III RCTs	Patients with *ESR1* mutations who have previously received ET showed a PFS benefit when treated with oral SERDs versus standard-of-care ET in the second or subsequent-line (hazard ratio 0.58; 95% CI 0.47–0.71; *p* < 0.00001), indicating that *ESR1* mutations are a marker of resistance to traditional ET and indicate patients who would benefit from treatment with oral SERDs [33]	I
	Retrospective analysis of two Phase III RCTs	Presence of *ESR1* mutation at baseline can predict lack of benefit from subsequent aromatase inhibitor treatment [34]	II
	Phase III RCT	Patients with rising *ESR1* mutation detected in ctDNA during first-line treatment with aromatase inhibitor + palbociclib showed significantly improved PFS following a switch to fulvestrant + palbociclib compared to those who remained on aromatase inhibitor + palbociclib (hazard ratio 0.61; 95% CI 0.43–0.86; *p* = 0.004) [35]	I
	Prospective study	*ESR1* mutations were associated with shorter PFS on CDK4/6 inhibitor + ET independently of ET resistance status (note: ET type not specified) [17]	III
	Prospective study	Patients with an *ESR1* mutation (n = 9) had a shorter PFS than those without, and *ESR1* mutation was an independent predictor of PFS; *ESR1* mutation status likely indicates patients who will be less responsive to ET with aromatase inhibitors [36]	III
	Retrospective study	*ESR1* mutations are less frequent in patients with primary ET resistance and <1 year of adjuvant ET than in patients with secondary ET resistance [37]	IV
	Retrospective analysis of Phase III RCT	*ESR1* mutations were more frequent after 6 months of first-line treatment with aromatase inhibitor + palbociclib and decreased in incidence after 3 years of treatment [38]	I
	Retrospective study	Baseline *ESR1* mutations were associated with worse survival outcomes in patients receiving first-line CDK4/6 inhibitor + aromatase inhibitor, but not in patients receiving CDK4/6 inhibitor + fulvestrant [39]	IV
*PIK3CA*/*AKT1*/*PTEN* alterations	Retrospective analysis of Phase II RCT	*PTEN* loss was associated with shorter PFS on CDK4/6 inhibitor + ET, indicating an elevated level of treatment resistance [40]	II
	Phase III RCT	Patients receiving capivasertib + fulvestrant in patients who also have *PIK3CA*/*AKT1*/*PTEN* mutations show statistically significantly improved PFS versus placebo + fulvestrant (hazard ratio 0.60; 95% CI 0.51–0.71; *p* < 0.001), indicating sensitivity driven through the combination partner [41]	I
	Phase III RCT	Patients receiving alpelisib + fulvestrant with a *PIK3CA* mutation showed improved PFS versus placebo + fulvestrant (hazard ratio 0.65; 95% CI 0.50–0.85; *p* < 0.001), indicating sensitivity driven through the combination partner [42]	I
	Phase III RCT	Patients receiving abemaciclib + fulvestrant following progression on ET showed improved PFS over placebo + fulvestrant, with numerically greater PFS benefit observed for patients with *PIK3CA* (hazard ratio 0.53; 95% CI 0.33–0.84) or *ESR1* mutations (hazard ratio 0.54; 95% CI 0.37–0.79) detectable in baseline ctDNA [43]	I
	Phase III RCT	Patients receiving inavolisib + palbociclib + fulvestrant with a *PIK3CA* mutation showed significantly longer PFS than placebo + palbociclib + fulvestrant (hazard ratio 0.43; 95% CI 0.32–0.59; *p* < 0.001) [44] and significantly improved OS (hazard ratio 0.67; 95% CI 0.48–0.94; *p* = 0.02)) [45]	I
*BRCA1*/*2* mutations	Retrospective study	BRCA mutations associated with shorter PFS and OS in patients receiving CDK4/6 inhibitor + ET [46]	IV
	Retrospective study	gBRCA mutation status is associated with a lower OS and PFS following ET monotherapy but not following single-line chemotherapy; gBRCA mutation status likely indicates that a patient will be less responsive to ET [47]	IV
	Retrospective study	Patients with BRCA mutation show a worse prognosis regardless of PAM50 subtype and luminal status, indicating BRCA mutations as a general marker for resistance [40]. It should be noted that this analysis, though significant, only included a limited sample of patients with BRCA mutations (N = 3; 4%)	IV
Rare genomic alterations			
*NTRK* fusion	Retrospective study	*NTRK* fusion was associated with progression on ET in patients and with promoting ET resistance in cell lines [48]	IV
*RET* mutations	Retrospective study	Enhanced *RET* expression was associated with worse prognosis and development of resistance to aromatase inhibitors in patients with estrogen receptor-positive invasive breast cancer (N = 52) [49]	IV
	Retrospective study	High *RET* expression was associated with worse outcomes, including PFS and OS, in patients with hormone receptor-positive tumors [50]	IV

ABC, advanced breast cancer; *AKT1*, Akt serine/threonine kinase 1; (g)BRCA, (germline) *BRCA1* and/or *BRCA2*; CDK4/6, cyclin-dependent kinase 4/6; CI, confidence interval; ESMO, European Society for Medical Oncology; *ESR1*, estrogen receptor 1; ET, endocrine therapy; HER2, human epidermal growth factor 2; HR, hormone receptor; *NTRK*, neurotrophic tyrosine receptor kinase; OS, overall survival; PFS, progression-free survival; *PIK3CA*, catalytic subunit alpha of phosphatidylinositol-3-kinase; *PTEN*, phosphatase and tensin homolog; RCT, randomized clinical trial; *RET*, rearranged during transfection; SERD, selective estrogen receptor degrader; TTF, time to treatment failure.

a I, evidence from at least one large randomized, controlled trial of good methodological quality (low potential for bias) or meta-analyses of well-conducted randomized trials without heterogeneity; II, small randomized trials or large randomized trials with a suspicion of bias (lower methodological quality) or meta-analyses of such trials or of trials with demonstrated heterogeneity; III, prospective cohort studies; IV, retrospective cohort studies or case–control studies; V studies without control group, case reports, expert opinions.

### 3.1 Estrogen receptor expression

Estrogen receptor status is a predictive marker for response to ET. Current ASCO/College of American Pathologists guidelines recommend that a tumor sample be interpreted as estrogen receptor-positive (and eligible for ET) if ≥ 10% of nuclei stain positive by immunohistochemistry (IHC), estrogen receptor-negative if <1% staining is observed, and estrogen receptor-low within the 1–10% range [55]. A second measure of estrogen receptor expression, the Allred score, is derived from combining the proportion of cells staining positive (scale of 0–5) with the intensity of staining (scale of 0–3), to result in a total score from 0 to 8. An Allred score ≥2 is interpreted as estrogen receptor-positive [56].

Higher estrogen receptor expression has been associated with better response and survival outcomes (Table 1) [[21], [22], [23]], while patients with tumors with low expression may benefit from treatments other than ET [57,58]. Differential levels of estrogen receptor expression within patients with HR-positive/HER2-positive ABC are not currently incorporated into guidelines as a marker to guide treatment decision-making [1,[3], [4], [5], [6]]. However, intrinsic subtyping evaluating subtypes defined by gene expression has provided convincing data that tumors with estrogen receptor expression <10% behave and respond more like triple-negative cancers [59], suggesting that treatment in this subgroup should be tailored by the degree of estrogen receptor expression and that this marker is strongly associated with the spectrum of ET suitability.

Estrogen receptor expression may change over time. Analysis of matched primary and metastatic tumors from patients with metastatic breast cancer across a number of studies have found that a change in estrogen receptor status from positive to negative was associated with poorer survival outcomes [[60], [61], [62], [63]]. ESMO Clinical Practice Guidelines recommend a biopsy for all patients with newly diagnosed or recurrent ABC to reassess estrogen receptor status (as well as progesterone receptor and HER2 status), except in cases where biopsy is not technically feasible, for example bone metastases where the decalcification process may limit biomarker detection; a patient should be considered ET suitable when the tumor has been recorded as estrogen receptor-positive in a prior biopsy, if a more recent biopsy is not possible [18]. A subsequent tumor biopsy may be considered to assess receptor status and reassess ET suitability if the tumor is not responding or behaving as expected from prior results.

### 3.2 Disease-free interval after prior ET

Current ABC guidelines for metastatic breast cancer suggest that patients with a long disease-free interval following adjuvant ET or longer duration on ET before relapse/disease progression are likely to be ET sensitive, whereas those progressing within 2 months on second-line or later-line ET are likely insensitive and would no longer benefit from further ET [4]. Supporting this, a retrospective study of 252 patients with metastatic breast cancer found that sensitivity to prior ET is a marker for sensitivity to fulvestrant; patients with a duration of ≥25 months on prior adjuvant ET and ≥5 months on prior first-line ET achieved a longer progression-free survival (PFS) on fulvestrant (Table 1) [24]. In this case, sensitivity and suitability are well-aligned; patients with a long disease-free interval are likely to be sensitive to, and suitable for, further lines of ET.

### 3.3 Visceral crisis

In guidelines that recommend chemotherapy over ET as first-line treatment for ABC, the presence of visceral crisis and imminent organ failure are key and long-standing criteria [[2], [3], [4], [5], [6],^14^], suggesting that they are markers for low ET suitability.

Approximately 15% of patients with ABC present with visceral crisis, although the term remains poorly defined [4]. A formal definition of visceral crisis was introduced in the 2nd ESO-ESMO international consensus guidelines for ABC (ABC2): severe organ dysfunction determined by signs and symptoms, laboratory evaluation, and rapid disease progression [64]. A later edition further refined the definition in liver as rapidly increasing bilirubin >1.5 times the upper limit of normal (ULN) [15]. Additional definitions of visceral crisis with other organ involvement have been proposed, although these definitions have not been specifically adopted more widely [65]. As such, the definition of visceral crisis substantially overlaps with the ‘imminent organ failure’, the latter of which is the preferred term used by the ESMO guidelines [66].

The definition of true visceral crisis and use of imminent organ failure as a key treatment decision point is based on early evidence that chemotherapy, particularly combination regimens, may elicit a faster and more robust response than ET [[67], [68], [69]]; in some settings there is evidence that supports an association between visceral relapse and ET resistance (Table 1) [16].

However, the availability and efficacy of ET + targeted agents such as CDK4/6 inhibitors has modified this approach; trials evaluating ET + targeted therapy versus chemotherapy with or without maintenance ET have demonstrated superior efficacy of ET with CDK4/6 inhibition compared with chemotherapy as primary treatment for aggressive disease (Table 1) [[26], [27], [28]]. The open-label, randomized, Phase II RIGHT Choice trial recruited pre-/peri-menopausal women with HR-positive/HER2-negative ABC, no prior systemic therapy for ABC, and clinically aggressive disease, defined as symptomatic visceral metastases, rapid disease progression, impending visceral compromise, or markedly symptomatic non-visceral disease [26]. Visceral crisis (defined per ABC3 as severe organ dysfunction as assessed by signs and symptoms, laboratory studies, and rapid progression of disease; excluding patients with liver metastases with 1.5x the ULN bilirubin levels, or with aspartate transaminase or alanine aminotransferase levels >5x the ULN) was reported in 47.7% of patients [26]. In the overall population, ribociclib + ET significantly improved the primary endpoint of PFS versus combination chemotherapy (21.8 versus 12.8 months, hazard ratio 0.61 [95% confidence interval (CI) 0.43–0.87], p = 0.003), with favorable tolerability. Similarly, in the open-label, randomized, Phase IV PADMA trial, in patients with previously untreated HR-positive/HER2-negative ABC and an indication for chemotherapy, palbociclib + ET demonstrated a statistically significant and clinically meaningful improvement in time to treatment failure (TTF) and PFS versus chemotherapy ± maintenance ET (median TTF 17.2 versus 6.1 months, hazard ratio 0.46 [95% CI 0.31–0.69] p < 0.001; median PFS 18.7 versus 7.8 months, hazard ratio 0.45 [95% CI 0.29–0.70] p < 0.001) [27]. Although this evidence should be caveated by inconsistent definitions of visceral crisis, it is clear that in some cases, ET-based regimens may be a suitable approach for patients in this setting.

Furthermore, in some cases, ET-based approaches may achieve a higher early response rate in patients with visceral disease versus chemotherapy. The open-label, randomized, Phase II ABIGAIL trial compared abemaciclib + ET with chemotherapy + maintenance ET as first-line treatment of patients with HR-positive/HER2-negative ABC with at least one feature of aggressive disease (defined as visceral metastases, grade 3 and/or negative progesterone receptor on primary tumor, lactate dehydrogenase (LDH) >1.5 times the ULN, and/or disease progression ≤36 months after completing adjuvant ET); visceral disease was reported in 64% of patients [28]. The 12-week overall response rate was 59% with abemaciclib + ET and 40% with chemotherapy + maintenance ET (odds ratio: 2.12 [95% CI 1.13–3.96]; p = 0.019). Initial data from the AMBRE trial also indicated that abemaciclib + ET significantly improved PFS versus chemotherapy in patients with a high visceral tumor burden (median PFS 13.9 versus 7.0 months, hazard ratio 0.67 [95% CI 0.46–0.98], p = 0.035); visceral burden was described in the trial as presence of ≥2 visceral sites, ≥3 lesions in one organ, or visceral disease with LDH > ULN [70].

It is important to note that the trials mentioned above included patients with an indication for chemotherapy that is not in most cases representative of the most recently published ABC guidelines’ definition of visceral crisis. These trials also included patients who may not have been in “true” visceral crisis. In line with this variability in patient population, a subgroup analysis of the population from the RIGHT Choice trial showed that the PFS benefit in patients with visceral crisis was less pronounced [26]. As such, clearly and consistently delineating visceral crisis is vital for appropriate treatment sequencing.

Overall, although patients with visceral crisis have historically represented a population with poor prognosis that benefit from an aggressive treatment approach such as chemotherapy and have therefore been unsuitable for ET, evidence from certain trials suggests that ET-based regimens may be suitable for some patients to achieve a rapid tumor response.

#### 3.3.1 Visceral metastases in the absence of visceral crisis

Visceral metastases may also occur in the absence of visceral crisis. The data on how this affects response to ET-based regimens are mixed. Some randomized clinical trials (RCTs) have shown that outcomes with ET-based regimens are comparable in subgroups with or without visceral disease [41,[71], [72], [73]]. However, one earlier meta-analysis of 14 RCTs found that outcomes of ET monotherapy as either first- or second-line treatment were specifically worse in patients with liver metastases (Table 1) [25]. A more recent meta-analysis of 41 RCTs from 2024 suggested that patients with visceral metastases derive less PFS benefit from first- or further-line CDK4/6 inhibitors + ET compared to the overall population, although a PFS benefit in these patients was still evident versus control treatment [74]. In line with these data, a subgroup analysis of patients from the Phase III SONIA trial demonstrated that those with visceral metastases derived less benefit from first-line CDK4/6 inhibitor + ET than patients without visceral disease (time from randomisation to second objective disease progression, or death hazard ratio 0.93 versus 0.80, respectively) [75], but response to treatment was still observed; notably, at final analysis a trend towards better overall survival in premenopausal patients who had received prior CDK4/6i was observed (hazard ratio 0.54 [95% CI 0.27–1.02]) [76].

As such, visceral metastases appear to attenuate ET response but do not negate the benefits of receiving ET-based treatment altogether, likely reflecting their impact on prognosis overall rather than on response to ET specifically. This suggests that ET may be a suitable option for patients with visceral metastases if a patient has other markers that favor ET suitability.

### 3.4 HER2 status

This article primarily focuses on the treatment of HR-positive/HER2-negative ABC. However, it would be remiss not to briefly consider several recent studies that have examined the response to ET-based therapy of tumors with HER2-low status. HER2-low status describes a subset of HER2-negative tumors with an IHC score 1+ or 2+/*in situ* hybridization (ISH) not amplified [77]. Current guidelines recommend that HER2 status (HER2-low/HER-zero [IHC score 0]) be used to inform treatment decisions following disease progression on at least one line of chemotherapy [[4], [5], [6]].

The limited available evidence suggests that patients with HER2-low status tumors may have poorer prognosis on ET-based regimens versus HER2-zero status in patients with HR-positive/HER2-negative ABC (Table 1) [29,30]. However, the emergence of novel ET therapies may result in new ET-based options for patients with HER2-low/ultralow ABC. For example, in the EMERALD trial, elacestrant was associated with prolonged PFS versus standard-of-care regardless of HER2-low status in a pre-selected patient population who had received at least 12 months of prior CDK4/6 inhibitor [31]. Furthermore, the addition of palbociclib to anti-HER2 therapy + ET in patients who are HER2-positive was found to improve PFS versus anti-HER2 + ET alone, suggesting that novel therapeutic combinations could extend the benefit of ET in certain subtypes [78] and increase the range of patients for whom ET is a suitable approach.

### 3.5 Genomic alterations

Gene alterations in specific pathways can affect cancer proliferation, survival outcomes, and response to ET. They are therefore important factors for treatment sequencing in clinical guidelines, especially in the second- and subsequent-line settings [[1], [2], [3], [4], [5], [6]]. The ESMO Precision Medicine Working Group recently reclassified *ESR1*, somatic *BRCA1/2* and *PALB2* as ESMO Scale for Clinical Actionability of molecular Targets (ESCAT) level IA, IIB and IIB, respectively, and included *PTEN* and *AKT* with an ESCAT of I/II. *ESR1* and *PIK3CA* mutations have an ESCAT of IA. Guidelines currently recommend screening for molecular alterations following disease progression on first-line ET for HR-positive/HER2-negative ABC in order to sequence use of appropriate ET with or without relevant targeted therapies [[1], [2], [3], [4], [5], [6]].

Alterations can be identified through next-generation sequencing (NGS) or polymerase chain reaction test using tumor tissue or blood samples in clinical settings [6]. The sensitivity of liquid biopsy profiling is dependent on sufficient tumor DNA shedding in blood [79]. NGS of HR-positive/HER2-negative ABC is now recommended as standard-of-care, with testing conducted after resistance to ET is observed, to optimize the chance of detecting *ESR1* mutations [80]. The applicability of specific detection techniques in clinical practice is beyond the scope of this review; considerations for medical oncologists when ordering tests for NGS have been outlined in more detail by the ESMO Precision Medicine Working Group [80]. However, one challenge to consider in using alterations to determine ET suitability is how accessible and practical their detection is in daily clinical practice.

Molecular alterations may identify patients at higher risk of early progression on ET, affecting their suitability for multiple lines of therapy over time. For example, retrospective analysis of patients treated with ET alone or in combination with CDK4/6 inhibitor showed the presence of gBRCA mutations was associated with poorer survival outcomes (Table 1) [40,46,47]. Conversely, combining targeted therapies with ET for patients who have certain actionable alterations (such as PI3K/AKT inhibitors and those targeting *ESR1* mutations) has been shown to improve outcomes in patients versus ET monotherapy (Table 1) [17,[32], [33], [34], [35], [36], [37], [38], [39], [40], [41], [42], [43], [44], [45]], thus broadening the population of patients suitable to receive further ET-based therapy. Some clinical trials have demonstrated the possible benefit of testing for emergent *ESR1* mutations through ctDNA monitoring while on first-line therapy to inform treatment switching before progression occurs, extending the duration of first-line ET [32,81].

It is clear from the existing evidence that both detection of specific alterations and also monitoring of their emergence over time are important factors in determining the suitability of a patient for ET, assuming that relevant tests are regionally available and accessible.

#### 3.5.1 Rare genomic alterations

Several rare alterations may also factor into treatment decisions and affect response to and suitability for ET in some patients. Neurotrophic tyrosine receptor kinase (*NTRK*) fusions, although rare in breast cancer (found in <1% of breast cancers [48]), have been associated with development of ET resistance. Fusion testing in ABC, particularly in patients whose disease progresses after ET, may benefit select patients who harbor targetable kinase fusions [48,82]. Oncogenic alterations in rearranged during transfection (*RET*) receptor tyrosine kinase are also rare in breast cancer (found in approximately 1% of breast cancers) [[83], [84], [85]]; RET and estrogen receptor pathways can functionally interact, potentially affecting the sensitivity of breast cancer cells to ET [86]. In addition to *in vitro* studies demonstrating that *RET* signaling can promote ET resistance [49,87], retrospective analyses have shown that elevated RET expression is associated with poor clinical outcomes and ET resistance (Table 1) [49,50]. Given the existing data, when considered alone these rare alterations reduce the suitability of implementing an ET-based regimen.

Although the above established biomarkers and clinical factors set important foundations to assess ET suitability in the clinic, the use of these in isolation may not accurately reflect population heterogeneity or the need for individualized treatment plans. Further investigation into several emerging markers, discussed below, may help to delineate a more holistic approach.

## 4 Potential role of emerging and investigational markers on suitability for ET

Several markers have emerged as potential contributors to ET sensitivity and/or suitability for specific endocrine-based regimens (Table 2) [17,36,40,88,[90], [91], [92], [93], [94], [95],[97], [98], [99], [100]]. Similar to more established markers, these can be overlaid onto the same 2D-framework (Fig. 2) that visualizes a marker's effect on ET sensitivity versus ET suitability, with the caveat that there is less available evidence to support their positioning and that more research is required before they can be brought into daily clinical practice.

**Table 2:** Emerging markers of ET suitability. Evidence strength graded using ESMO methodology.

Marker	Study type	Key evidence to support a role in influencing ET suitability	Evidence strength^a^
ctDNA concentration	Prospective study	• Patients with high cfDNA concentrations (≥2.6 ng/μL of plasma) had worse survival outcomes than those with low concentrations (<2.6 ng/μL) [36]	III
	Retrospective study of Phase III RCTs	Patients with low (<1%) ctDNA at baseline had a more favorable best overall response to treatment and also had more favorable prognostic factors than patients with high (≥1%) ctDNA at baseline [88]	II
	Exploratory analysis of Phase II RCT	Patients with rapid progression had significantly higher baseline ctDNA levels than patients without rapid progression [89]	II
CTC count	Phase III RCT	CTC count may be a reliable biomarker for guiding the decision between ET and chemotherapy: following a pattern of assigning patients with <5/7.5 mL to ET and ≥5/7.5 mL to chemotherapy, no overt survival differences were identified between the two study arms (hazard ratio 0.94; 90% CI 0.81–1.09) [90]	I
	Retrospective study	Patients with a CTC count ≥2 have worse PFS on ET versus chemotherapy; these differences are not found at CTC counts <2, indicating that patients with a lower CTC are ET sensitive, whereas those with a higher CTC may be ET resistant [91]	IV
Baseline sTK1 levels	Retrospective analysis of Phase III RCT	High sTK1 levels at baseline are associated with worse PFS (hazard ratio 1.76; 95% CI 1.43–2.16; *p* < 0.0001) and OS outcomes (hazard ratio 2.38; 95% CI 1.91–2.98; *p* < 0.0001) in patients with ABC starting first-line ET; low sTK1 levels may be used to identify patients who may benefit from ET [92]	II
	Phase III RCTs	High pretreatment sTK1 in the first-line setting and beyond may be an independent prognostic factor for worse survival outcomes [93,94]	I
*TP53* mutations	Exploratory analysis of Phase II RCT	Patients receiving palbociclib + ET exhibited worse PFS outcomes in the presence of baseline *TP53* mutations [40]	II
	Prospective study	*TP53* mutations were associated with worse PFS outcomes in patients treated with first-line CDK4/6 inhibitor + ET (N = 411) [17]	III
	Prospective study	*TP53* alterations were associated with worse PFS and OS in patients with HR-positive/HER2-negative ABC receiving first-line therapy, the majority of whom had previously received adjuvant ET [95]	III
	Prospective study	*TP53* mutations were associated with shorter recurrence-free survival, PFS and overall survival [96]	III
*RB1* mutations	Phase III RCT	*RB1* mutations emerged in a minority of patients (N = 6; 4.7%) treated with palbociclib + fulvestrant (but not in patients treated with placebo + fulvestrant) [97]	I
	Phase II RCT	In patients treated with ET in the second or subsequent-line (N = 293), baseline *RB1* mutations were associated with rapid progression (PFS <3 months) both in the overall population and in patients with *ESR1* mutations at baseline [98]	II
	Exploratory analysis of Phase II RCT	Patients receiving palbociclib + ET (N = 109) exhibited worse PFS outcomes in the presence of baseline *RB1* pathway alterations (hazard ratio 2.62; *p* = 0.06; n = 4) [40]	II
	Prospective study	*RB1* mutations were associated with worse OS in patients with HR-positive/HER2-negative ABC receiving first-line therapy (n = 63), the majority of whom had previously received adjuvant ET [95]	III

ABC, advanced breast cancer; *BRCA2*, breast cancer gene 2; CDK4/6, cyclin-dependent kinase 4/6; cfDNA, circulating free DNA; CTC, circulating tumor content; ctDNA, circulating tumor DNA; ESMO, European Society for Medical Oncology; ET, endocrine therapy; HER2, human epidermal growth factor receptor 2; HR, hormone receptor; OS, overall survival; PFS, progression-free survival; *RB1*, retinoblastoma 1; RCT, randomized clinical trial; sTK1, serum thymidine kinase 1; *TP53*, tumor protein P53.

a Ievidence from at least one large randomized, controlled trial of good methodological quality (low potential for bias) or meta-analyses of well-conducted randomized trials without heterogeneity; II, small randomized trials or large randomized trials with a suspicion of bias (lower methodological quality) or meta-analyses of such trials or of trials with demonstrated heterogeneity; III, prospective cohort studies; IV, retrospective cohort studies or case–control studies; V studies without control group, case reports, expert opinions.

Markers that indicate a higher tumor burden and/or activity could impact response to anticancer therapy and, hence, suitability for ET. For instance, in the acelERA Phase II trial patients with significantly higher plasma circulating tumor DNA (ctDNA) levels at baseline were more likely to have rapid progression (PFS <3 months) while on giredestrant or physician's choice of ET versus patients with lower ctDNA levels at baseline [89]. In line with these findings, an exploratory pooled analysis of baseline ctDNA levels in patients from the MONALEESA Phase III trials found that patients with low (<1%) ctDNA fraction had a more favorable best overall response to treatment with CDK4/6 inhibitor (ribociclib) with ET than patients with high (≥1%) ctDNA levels at baseline [88]. Importantly, ctDNA levels are a standalone prognostic marker for disease prognosis and risk of recurrence in early breast cancer [101]; as such, it is not only linked to ET response but also the suitability of a patient for ET in the broader context of their disease severity. Taken together, this suggests that an individual's ctDNA fraction should be considered as part of a patient's ET suitability assessment, with lower ctDNA fractions adding weight in favor of further ET-based regimens.

Circulating tumor cell (CTC) count has also been implicated in response to ET; patients with higher CTC counts show worse response to ET versus chemotherapy [90,91]. The Phase III STIC trial evaluated the utility of using CTC counts to inform whether patients should receive ET or chemotherapy; improved PFS was observed in the CTC-informed treatment arm compared with the investigator-led control arm (15.5 months versus 13.9 months, respectively), suggesting that considering CTC count has some benefit when determining next-line therapy [90].

Evidence suggests that *TP53* [17,40,95,96] and *RB1* alterations [40,95,97,98] are associated with poor outcomes and development of resistance while on ET-based treatment. Although not particularly common in metastatic breast cancer [102], the presence of *ERBB2* mutations may also be associated with poor response to endocrine therapy [103]. Furthermore, co-occurrence of certain alterations can compound the impact on treatment response. For example, analysis has shown that g*BRCA2-*mutated tumors (discussed in a previous section) are also enriched for *RB1* loss-of-function mutations, driven by g*BRCA2* homologous recombination deficiency and *RB1* loss of heterozygosity status, resulting in poor outcomes on ET + CDK4/6 inhibitor [104]. High pretreatment serum thymidine kinase 1 levels before first- or subsequent-line ET also appears to be an independent prognostic factor for worse survival outcomes. However, many of these markers are rarely tested or reported in daily clinical practice, and more work is needed to establish the practicality of assessing these marker in the clinic [[92], [93], [94]]. As such, while they have a known impact on ET sensitivity, they do not yet have a notable role in determining ET suitability.

Another emerging marker that may support second-line treatment selection is [^18^F]fluoroestradiol (FES) uptake, assessed by positron emission tomography or computed tomography imaging. FES uptake in tumor cells has been shown to work as a proxy for estrogen receptor expression [105] and has been used to assess *in vivo* pharmacodynamics of different endocrine therapies, such as fulvestrant [106]. As such, the differential uptake of FES across tumor tissues could be used to identify patients who may be suitable for second- or further-line ET [107,108].

Although the markers discussed above are not regularly investigated in daily clinical practice, further research could establish feasibility of use in conjunction with established biomarkers and clinical factors and thus as determinants of ET suitability.

## 5 Considerations for treatment sequencing

Ongoing research has focused on how clinical characteristics and biomarkers may interact with treatment response, naturally raising the question of how oncologists should consider such markers when determining treatment sequencing. It is clear that no single marker can or should be used in isolation to determine ET suitability. Instead, a combination of clinical factors (including patient characteristics, disease course, and previous response) and tumor characteristics and biomarkers must be used to support clinical decision-making.

There are several key considerations specific to treatment sequencing. First is the impact of prior therapy on ET suitability and treatment sequencing, comprising: treatment-free interval after adjuvant ET, response to prior ET in the metastatic setting, prior CDK4/6 inhibitor duration, number of lines of prior ET, choice of prior therapy, prior chemotherapy in the advanced stage setting, and tolerability of prior ET. Second is the relative importance of established and emerging clinical markers and biomarkers, the most important clinical markers of which are: organ function, symptoms and symptom burden, the presence of visceral disease, speed of progression, comorbidities, and treatment tolerance; and most important biomarkers of which are: estrogen receptor expression*, ESR1* mutations, mutations/alterations in *PIK3CA/AKT1/PTEN*, HER2 low/ultralow*, BRCA1/2* and *PALB2* mutations.

These characteristics, and others depending on the individual patient and patient's tumor biology, influence the decisions regarding the appropriate sequencing of therapy both with ET and targeted agents and therefore how ET suitability may change with each line of therapy. It is the opinion of this expert author group that sequential ET with targeted agents should be the standard-of-care in patients with endocrine responsive, more indolent disease with low symptom burden, with line of therapy impacting the relative importance of symptoms, extent of organ involvement, duration of prior response, and organ function. Conversely, patients with rapid disease progression, substantial symptom burden, and impending visceral crisis/true visceral crisis are not likely to be suitable for ET.

In addition, it is important to note that tumor characteristics may change over time, with loss of hormone receptor expression and even appearance of expression discordant with results in the early-stage setting [63]. As such, a patient who was determined to be suitable for ET in prior assessments may not be suitable for further ET upon disease progression (or *vice versa*). There is, therefore, an urgent need for tailored, adaptable treatment pathways that can flex according to changing individual needs. Despite this, in current practice, patients are not typically retested for change in markers, which may result in suboptimal treatment selection. The decision about when to test for a change in markers after the initial diagnosis of metastatic disease should be driven by unexpected or less-than-expected response to ET, and where results will impact therapeutic decisions.

## 6 Guidance for future research

At present, clinical characteristics that can be readily assessed in day-to-day practice remain of paramount importance in clinical decision-making. Based on the currently available data, it remains challenging to translate the level of ET resistance as defined by markers into a clinically meaningful likelihood of benefit from treatment. There is a clear need to deepen understanding of the current research and delineate how it can be applied to daily clinical decision-making.

A key shift in approach would be moving the focus of research from ET sensitivity to ET suitability; these two concepts are linked, with suitability being more relevant to clinical practice​. Although sensitivity and suitability for ET may often correspond, in some cases, patients who are ET sensitive are not suitable for further ET for other reasons; for example, a patient who is ET-naïve but with imminent organ failure. *Vice versa*​, some patients may be suitable for further ET despite displaying some level of resistance to specific ET-based therapies; for example, patients with *ESR1* mutations may benefit from next-generation SERDs, but would not be suitable for further treatment with aromatase inhibitors. This is especially true in the combinatorial approach setting, and when considering differences in the availability of treatment by region. Furthermore, patient preference and overall goals of care must be considered.

Ongoing research may establish new pathways for treatment selection; a clear outstanding need is for research that can integrate multiple markers of suitability and/or response into an actionable model. Machine-learning algorithms are increasingly being explored as a way of supporting treatment decisions [109]. One such algorithm used a combination of clinical and genomic information to stratify patients into risk groups based on predicted PFS [110], and is a clear example of how emerging technologies could be used to identify ET suitable patients. Development of a validated algorithm focusing on key markers such as presence of visceral metastases, time on prior CDK4/6 inhibitor, time on second-line ET (if given), genomic alterations, germline mutations, and HER2-ultralow/-low status, may establish a unified baseline for future decision-making. It is critical that biomarkers and clinical characteristics are considered in combination and not as single factors.

In addition to considering individual versus holistic assessment of markers, several practicalities must be accounted for when determining how suitable a patient is for further ET. For example, the regional availability of different ET options must be considered as not all regions​ have access to the same ET options; for example, in certain regions a patient may be likely to respond well to specific ET-based regimens but unsuitable for ET given availability of ET-based therapies in their place of treatment. Furthermore, the practicality of measuring and monitoring markers in regular clinical practice must be assessed, as many assays are not conducted as part of typical monitoring and/or may require specialized, costly equipment.

There remain several outstanding questions in the field, including how we can address tumor heterogeneity, what the most appropriate sequencing of endocrine and targeted agents is, what degree of precision in marker assessment is required to understand the benefit of ET for an individual patient, better understanding of the biology underlying resistance mechanisms, what threshold for biomarker expression makes it worthwhile (or not) to proceed with ET, and how should patient preference interface with treatment selection. Furthermore, we must consider whether there are situations where re-challenging with ET is appropriate after chemotherapy (i.e., following an antibody–drug conjugate), if there is a role for combining ET and chemotherapy, and whether ET can be used effectively as maintenance after chemotherapy induction in situations of true visceral crisis.

Further work and ongoing studies will help to support the evolution of guidelines alongside the introduction of new treatments for HR-positive/HER2-negative ABC, as clear and comprehensive recommendations are critical in this fast-moving, heterogenous disease and treatment landscape.

## CRediT authorship contribution statement

**Hope S. Rugo:** Conceptualization, Writing – original draft, Writing – review & editing. **Giuseppe Curigliano:** Conceptualization, Writing – original draft, Writing – review & editing. **David W. Cescon:** Conceptualization, Writing – original draft, Writing – review & editing. **Frédérique Penault-Llorca:** Conceptualization, Writing – original draft, Writing – review & editing. **Nadia Harbeck:** Conceptualization, Writing – original draft, Writing – review & editing. **Seock-Ah Im:** Conceptualization, Writing – original draft, Writing – review & editing. **Yeon Hee Park:** Conceptualization, Writing – original draft, Writing – review & editing. **Carlos Barrios:** Conceptualization, Writing – original draft, Writing – review & editing. **Shanu Modi:** Conceptualization, Writing – original draft, Writing – review & editing. **Sara M. Tolaney:** Conceptualization, Writing – original draft, Writing – review & editing. **Mafalda Oliveira:** Conceptualization, Writing – original draft, Writing – review & editing.

## Declaration of competing interest

The authors declare the following financial interests/personal relationships which may be considered as potential competing interests: Hope S. Rugo reports article publishing charges and writing assistance were provided by AstraZeneca. Giuseppe Curigliano reports article publishing charges and writing assistance were provided by AstraZeneca. David W. Cescon reports article publishing charges and writing assistance were provided by AstraZeneca. Frederique Penault Llorca reports article publishing charges and writing assistance were provided by AstraZeneca. Nadia Harbeck reports article publishing charges and writing assistance were provided by AstraZeneca. Carlos Barrios reports article publishing charges and writing assistance were provided by AstraZeneca. Shanu Modi reports article publishing charges and writing assistance were provided by AstraZeneca. Sara M. Tolaney reports article publishing charges and writing assistance were provided by AstraZeneca. Mafalda Oliveira reports article publishing charges and writing assistance were provided by AstraZeneca. Seock-Ah Im reports article publishing charges and writing assistance were provided by AstraZeneca. Yeon Hee Park reports article publishing charges and writing assistance were provided by AstraZeneca. The authors meet criteria for authorship as recommended by the International Committee of Medical Journal Editors (ICMJE) and did not receive payment related to the development of this abstract. AstraZeneca was given the opportunity to review the manuscript for medical and scientific accuracy, as well as intellectual property considerations. Given their role as Editors, Hope Rugo, Frederique Penault-Llorca, Mafalda Oliveira and Seock-Ah Im had no involvement in the peer-review of this article and has no access to information regarding its peer-review. Full responsibility for the editorial process for this article was delegated to another journal editor. If there are other authors, they declare that they have no known competing financial interests or personal relationships that could have appeared to influence the work reported in this paper.
